# Notes and News

**Published:** 1898-02-05

**Authors:** 


### Notes and News.

(Collected and Communicated.)

HOSPITAL MEETINGS.

Royal National Hospital for Consumption, Ventnor.—Annual general meeting, Wednesday, February 9th, 4 p.m., at 34, Craven Street, Charing Cross.

North London Hospital for Consumption.—A festival dinner, in aid of this hospital, is to be held on May 19th, at the Hotel Cecil.

The Mayor of King's Lynn is appealing for funds towards the relief of sufferers through the recent typhoid epidemic.

An admirable new hospital is now being erected on the site of the picturesque old Ospedale Italiano in Queen Square, Bloomsbury.

The Willesden Cottage Hospital, by the assistance of Mr. Passmore Edwards, is to be extended at a cost of £2,500, and will in future be known as the "Passmore Edwards" Hospital for Willesden.

The treasurer of Guy's Hospital has received from Mr. Henry Lewis Raphael the sum of £20,000 to be devoted to the building of the "Henrietta Raphael Nurses' Home," in memory of the late Mrs. Raphael.

The seventeenth Congress and Exhibition of the Sanitary Institute will be held in September at Birmingham. H.R.H. the Duke of Cambridge will preside at the forthcoming Sanitary Institute dinner on March 23rd.

Five Russian physicians have been sent to India to watch the progress of the plague, and Mohammedan pilgrims have been prohibited from leaving Russian territory, lest they might import the epidemic on their return.

Mr. Herbert Allingham, F.R.C.S., has been appointed surgeon to H.R.H. the Prince of Wales's household.

Cases of rabies having occurred in the neighbourhood of Brighton, a pack of beagles and about 180 other dogs have been destroyed during the past week. The muzzling order has also been extended over portions of Kent.

New operating-rooms have just been completed at the North Staffordshire Infirmary, with an excellent nurse's store and sterilising-room adjoining, the whole being fitted with terrazza floors, glass shelves, and every up-to-date antiseptic improvement.

Notables whose autographs are in demand may take a hint from a popular actor and actress, who send a printed form to applicants for autographs, promising that these will be sent on receipt of a Jubilee stamp—an admirable method of swelling the Jubilee Hospital Fund.

Mr. Braxton Hicks opened an inquest last week at Battersea on two children who were said to have died from the effects of vaccination. It transpired that a third child had died that morning from erysipelas following vaccination by the same lymph, and that four or five other children were suffering from erysipelas from the same cause. The inquiry was adjourned.

The Indian Government have decided to place at the disposal of the Bombay Plague Committee the Durham and Shropshire Regiments to assist in the inspection of houses. An engineer officer and a civilian have also been appointed to strengthen the committee. The Bombay authorities have telegraphed to Dr. Lustig, the Italian professor of pathology who went to India last year to study the bubonic plague, for a large supply of his anti-serum, which is said to have produced satisfactory results.

Dr. A. R. Cook, of the Church Missionary Society, rendered effectual help during the recent mutinies in Uganda. Writing of his surgical work among the wounded, he says: "A man was brought to me with a bullet in his leg. I cut down on it, and with a little trouble extracted it, and found a large Snider bullet. The man had been shot in the Mohammedan War at Mengo in 1888, and had been carrying the bullet for nine years. He found it got in his way when kneeling down to aim. When I had extracted it he begged for it to put in his gun, and to fire it in revenge at the Nubians. I need hardly say I have kept such an interesting relic."

Mr. D'Eyncourt gave a decision of great importance, both to milk dealers and their customers, at the North London Police-court on January 19th, to the effect that he could not convict a milk carrier detected in adding three pints of water to the milk he was entrusted by his employer in South Hornsey to deliver to his customers on the ground that it was done without malice. It was, however, shown that the milk so adulterated was thereby damaged, compelling the owner to throw it away, while had a Government inspector happened to have taken a sample of the milk so treated the owner would most certainly have been fined £20. It is satisfactory to know that the case will probably be taken to a superior Court, for if allowed to remain under the present decision it is a premium to milk carriers to defraud the public and injure their masters.

